# Systems consultation: protocol for a novel implementation strategy designed to promote evidence-based practice in primary care

**DOI:** 10.1186/s12961-016-0079-2

**Published:** 2016-01-27

**Authors:** Andrew Quanbeck, Randall T Brown, Aleksandra E Zgierska, Roberta A Johnson, James M Robinson, Nora Jacobson

**Affiliations:** Center for Health Enhancement Systems Studies, University of Wisconsin-Madison, 1513 University Ave., Madison, WI 53706 USA; School of Medicine and Public Health, University of Wisconsin-Madison, Room 3832, 1100 Delaplaine Ct, Madison, WI 53715 USA; Center for Health Systems Research & Analysis, University of Wisconsin-Madison, 1109c Warf Office Building, 610 Walnut St., Madison, WI 53726 USA; School of Nursing, University of Wisconsin-Madison, 5130 Cooper Hall, Signe Skott; 701 Highland Ave, Madison, WI 53705 USA

**Keywords:** Clinical guideline adoption, Implementation strategies, Systems engineering

## Abstract

**Background:**

Adoption of evidence-based practices takes place at a glacial place in healthcare. This research will pilot test an innovative implementation strategy – systems consultation –intended to speed the adoption of evidence-based practice in primary care. The strategy is based on tenets of systems engineering and has been extensively tested in addiction treatment. Three innovations have been included in the strategy – translation of a clinical practice guideline into a checklist-based implementation guide, the use of physician peer coaches (‘systems consultants’) to help clinics implement the guide, and a focus on reducing variation in practices across prescribers and clinics. The implementation strategy will be applied to improving opioid prescribing practices in primary care, which may help ultimately mitigate the increasing prevalence of opioid abuse and addiction.

**Methods/Design:**

The pilot test will compare four intervention clinics to four control clinics in a matched-pairs design. A leading clinical guideline for opioid prescribing has been translated into a checklist-based implementation guide in a systematic process that involved experts who wrote the guideline in consultation with implementation experts and primary care physicians. Two physicians with expertise in family and addiction medicine are serving as the systems consultants. Each systems consultant will guide two intervention clinics, using two site visits and follow-up communication by phone and email, to implement the translated guideline. Mixed methods will be used to test the feasibility, acceptability, and preliminary effectiveness of the implementation strategy in an evaluation that meets standards for ‘fully developed use’ of the RE-AIM framework (Reach, Effectiveness, Adoption, Implementation, Maintenance). The clinic will be the primary unit of analysis.

**Discussion:**

The systems consultation implementation strategy is intended to generalize to the adoption of other clinical guidelines. This pilot test is intended to prepare for a large randomized clinical trial that will test the strategy against other implementation strategies, such as audit/feedback and academic detailing, used to close the gap between knowledge and practice. The systems consultation approach has the potential to shorten the famously long time it takes to implement evidence-based practices and clinical guidelines in healthcare.

## Background

### Context

This project addresses the urgent need to promote the adoption of evidence-based practices (EBPs) in healthcare by pilot-testing an innovative implementation strategy named the Systems Consultation Strategy (SCS). The SCS is based on an evidence-based quality improvement approach with roots in systems engineering. This approach, named NIATx (the Network for the Improvement of Addiction Treatment) [1-3], has been widely tested in addiction treatment. The SCS extends elements of the NIATx approach to reducing variation in opioid prescribing in primary care. The proposed approach is intended to be a generalizable approach to EBP adoption, used in this proposal for a specific problem (opioid prescribing practices) and setting (primary care).

The standard approach to improving medical practice includes developing and disseminating clinical guidelines. Developing the guidelines involves panels of experts systematically reviewing the literature, achieving consensus, and publishing the results in a medical journal intended for the clinical audience [4]; this approach leaves a great gap between clinical knowledge and clinical practice [5-8]. Various approaches have been tried to narrow this gap, such as providing educational materials, audit/feedback [9], and academic detailing [10], with mixed success; about 30–40% of patients do not receive evidence-based care, and about 20–25% of care given is unnecessary or potentially harmful [8, 11]. Clinicians tend to continue to do what is comfortable, and value personal experience and familiar practice routines over scientific evidence [12].

### Rationale for proposed pilot study

This protocol was funded under the United States National Institute of Health’s R34 funding mechanism, the purpose of which is to “*provide support for the initial development of a clinical trial or research project*.” In this case, the funding will support a pilot study of the SCS to prepare for a large randomized control trial that will test the SCS against alternative approaches to EBP adoption in primary care. The SCS (1) teams clinical guideline writers with implementation specialists and primary care physicians to translate guidelines into a checklist-based implementation guide; (2) selects, trains, and deploys physician peer coaches (systems consultants) to help primary care clinicians implement EBPs using evidence-based tools of systems engineering; and (3) focuses on process as a cause of variation in outcomes. Although the SCS is rooted in established theory and empirical research, the approach has not been formally tested. This pilot test is intended to answer questions about the feasibility, acceptability, and preliminary effectiveness of the approach by studying adaptations that will be made to tailor the NIATx approach to primary care. In the future randomized trial, we will assess the costs and effects of the SCS versus other implementation strategies (such as audit/feedback or academic detailing) to determine the most cost-effective approach for reducing variation in opioid prescribing practices.

Primary care was chosen as the setting because, at a broad level, a strategy for improving the adoption of EBPs in primary care could apply to diverse patient populations and outcomes, and because primary care physicians are the main prescribers of opioids [13]. If clinical practice can be changed by simply informing physicians of their opioid prescribing levels and how they compare to peers, then audit/feedback may suffice to reduce variation in opioid prescribing. If education beyond audit/feedback is required, nurses or other healthcare professionals may deliver it relatively inexpensively through academic detailing visits. However, evidence from prior quality improvement research [1] and theory on organizational and individual change [14,15] suggests that changing clinical practice may require a more comprehensive strategy.

### The prescription opioid crisis

This research focuses on a specific clinical practice in need of change – opioid prescribing for chronic pain. Opioid analgesics have been increasingly used to treat chronic non-cancer pain [16], despite mounting evidence of their adverse effects [17]. In the past, physician education encouraged physicians to listen to and treat patients’ pain complaints and (1) held that addiction was rare when pain medications were taken as prescribed, and (2) said that, if subjectively well-tolerated, opioids did not cause end-organ damage, and, hence, no ceiling [18] existed for dose increases [19,20]. These two tenets have been accompanied by alarming increases in the prescribing of opioids, along with prescription opioid misuse, addiction, and diversion. Prescribing in daily doses exceeding the equivalent of 100–120 mg of morphine equivalent daily dose (MEDD) is now associated with increased risk of addiction and overdose [21-23].

The increase in opioid-related harms in the past two decades has led to efforts to curb prescription opioid-related harms [24,25]. Although examples of positive change in opioid prescribing can be found in the literature, few (if any) approaches have been systematically studied using experimental design as proposed in the long-term strategy described here. The literature on practice change in healthcare has repeatedly shown that system-level changes occur at a glacial pace [26]. This research seeks to promote system-level change by pilot-testing a pioneering systems engineering approach to improving opioid prescribing practices in primary care.

### NIATx and NIATx 200

Since 2003, NIATx has been addressing quality issues in addiction treatment using a model based on evidence-based principles of organizational change [27]. Early evaluation showed the effectiveness of the NIATx model on reducing waiting time and increasing early engagement [2,3]. More than 3,500 organizations in the specialty addiction treatment field have implemented the NIATx model. In 2007, the National Institute on Drug Abuse funded a cluster-randomized trial called NIATx 200 that was designed to identify the “active ingredient” in the NIATx model. NIATx 200 (5 R01 DA020832-05) was among the largest trials of its kind ever conducted in healthcare. Of the approaches tested, namely interest circle calls (group teleconferences), learning sessions (large in-person meetings), coaching (one-on-one consulting with experts), and the combination of all three, coaching emerged as the most cost-effective for reducing patient waiting time and increasing clinics’ annual number of admissions.

Coaches in NIATx 200 helped clinics improve access through an initial site visit and regular email and phone communication with each clinic’s designated change leader and other clinic staff members. Coaches helped clinics conduct a walkthrough exercise and interpret its results; assess workflows and identify opportunities for improvement using systems-engineering tools such as flowcharting and the nominal group technique [28]; and implement changes using Plan-Do-Study-Act cycles [29].

Although individual coaching sounds expensive, it proved more effective and substantially less expensive than learning sessions (periodic, large face-to-face meetings that are common in healthcare improvement efforts) in the NIATx 200 study. The costs associated with convening healthcare professionals from various locations (lodging, food, mileage, etc.) add up quickly. Sending a qualified coach to visit a clinic and to provide other consulting via phone and email proved less expensive and more effective than learning sessions. Although coaching has been well tested in the specialty addiction field, the approach must be adapted to fit within primary care [30]. Early feedback showed that physicians had negative associations with the term ‘coaching’, so the term has been replaced by ‘consulting', the more familiar term to physicians.

### Evidence gap/opportunity

This proposal introduces an implementation strategy distinguished by several innovations: (1) a systematic process for clinical guideline translation; (2) a peer-to-peer consulting intervention that integrates relevant theory with empirical research on organizational change [1,14,15]; and (3) an explicit focus on process as a cause of variation in outcomes [29]. The SCS consists of a series of generalizable steps, including setting the larger context (patient safety); teaming clinical guideline writers with implementation specialists and clinical practitioners to distill the essence of the guideline into a succinct, checklist-based implementation guide; providing an outside systems consultant (i.e. a coach) with clinical expertise and experience; actively involving clinical staff (including primary care physicians, nurses, and physician assistants) in the implementation process; giving participants the ability to customize interventions (rather than using a one-size-fits-all approach); and providing tools, such as the walkthrough exercise, Plan-Do-Study-Act change cycles, the nominal group technique, and flowcharting, that promote problem-solving and rapid, incremental improvements.

### Theoretical foundations

The SCS rests on established theories of organizational change from systems engineering [29,31] and diffusion of innovations [14], as well as recent empirical research on quality improvement [1]. Deming established a number of canonical systems engineering principles in his influential book, *Out of the Crisis* [29]. Of these principles, the following are most important for the current protocol: (1) quality problems are almost always caused by poor processes and systems, and thus lie outside the purview of individuals to change; improving quality requires systemic approaches rather than singling out individuals, and (2) measuring and understanding process variation is critical to improving quality. The goal of the SCS is to reduce variability in opioid prescribing practices.

Even though a clinical guideline for opioid prescribing has been in place for several years [32], most clinicians do not make adoption decisions based solely on scientific evidence. Rogers’s work on diffusion of innovations [14] provides a theoretical framework for understanding why some ideas diffuse throughout systems while others do not. Although characteristics of ideas are important in diffusion, social factors largely supersede them. People (and organizations) are more likely to adopt an idea if they know of others similar to themselves who have done so already. Diffusion of innovations theory stresses homophily between change agents and those whose behaviour they seek to influence, that is, the similarity between individuals on characteristics such as education and social status, suggesting that consulting intended to affect physician prescribing practices should be done by consultants who are physicians.

The idea of systems consulting rests on self-determination theory [15]. The theory holds that satisfying three fundamental needs (competence, relatedness, and autonomy) contributes to individual high functioning. Conditions that foster meeting these needs lead individuals to move from extrinsic to intrinsic motivation – a change that’s necessary for organizational improvement efforts to succeed. According to self-determination theory, extrinsic motivation encourages people to regard the cause of their actions as being outside themselves rather than thinking of themselves as agents of change. Extrinsic motivators (e.g. tangible rewards, deadlines, threats, directives, imposed goals) have all been shown to reduce intrinsic motivation [15]. In terms of opioid prescribing, an extrinsic motivator for physicians would be the threat of additional regulation. Self-determination theory posits that behaviour can become self-determined, or intrinsically motivated, if actions are modelled or valued by others to whom an individual feels related or attached. People are more likely to adopt actions when they feel competent doing the activities, and to adopt changes that relate to their goals and values.

### Contribution of this research

The SCS offers two innovations to promote the adoption of EBPs. First, panels of experts are commonly convened to reach consensus and develop guidelines for EBPs, as was done for opioid prescribing [32]. This proposal adds a novel step by bringing together guideline writers, implementation experts, and primary care physicians to translate the guideline into a checklist-based format that can be implemented more easily than a guideline appearing in an academic journal. The experts convened for this proposal include pain physicians from the panel that developed a leading opioid prescribing guideline; internationally recognized experts on healthcare quality improvement and drug policy; and community-based family medicine physicians (see Acknowledgments). Collectively, the advisory panel monitors and advises the research team throughout the pilot test of the implementation strategy.

Second, peer-to-peer physician consulting is a cornerstone of the SCS. While many efforts have been made to increase the use of EBPs among physicians, a literature review revealed a surprising dearth of research on models in which physicians consulted other physicians in primary care. Medical conferences have been used to promote new practices, but they are generally not effective [33]. Improvement collaboratives are a common method in healthcare; these involve clinical teams (which often include physicians and other clinicians) learning with and from one another [34]. More targeted types of physician education (such as coaching, facilitation, and academic detailing) often are conducted by health professionals such as nurses, physician assistants, or others with master’s degrees in business administration, public health, counselling, public administration, or social work [9,35-37]. In our literature review, the Physicians’ Clinical Support System − Buprenorphine came close to offering a formal physician-to-physician consulting model [38], though it did not include on-site visits and implementation support, and little program evaluation took place beyond statistics on the frequency and type of contact with mentors. Dartmouth Medical School developed a consulting model for medical students in which they received guidance and career development advice from senior faculty members [39], a model that took place only in an academic setting and did not target clinical practice.

## Methods/Design

### Preliminary studies

In preparation for the grant submission, we analyzed preliminary data from the set of family medicine clinics that form the recruitment base for this pilot study. Substantial differences in opioid prescribing practices were evident. Clinics, and providers within clinics, varied substantially in the percentage of patients with three or more opioid prescriptions in the past year and average MEDD per patient. Compared with other patients, those with three or more opioid prescriptions more frequently reported smoking and drinking, were diagnosed more often with depression, and had substantially more clinic visits. Those in the highest MEDD group (4th quartile) had the least favourable profile. Of note, close to 50% of those in the 4th MEDD quartile reported regular alcohol consumption, which is discouraged in treatment agreements. These preliminary results align with evidence on the dose–response relation between MEDD and poor outcomes, [40] and further suggest that implementing universal precautions for opioid prescribing can reduce harms.

Specific Aim 1 consists of guideline translation and training of the systems consultants. Guideline translation has been conducted by the research team in consultation with the advisory panel. We followed a structured group decision-making approach called the integrative group process [41], a systematic technique for facilitating meetings of experts that incorporates the nominal group technique [28], the Delphi process [42], social judgment analysis [43], and cognitive mapping [44]. We began by conducting a structured Delphi process [28], in which we asked each member of the advisory panel to rate each recommendation in the opioid prescribing clinical guideline on its measurability, potential to reduce opioid abuse, and ease of implementation. We conducted follow-up telephone interviews with each panel member to understand the ratings they assigned. We compiled the results of the Delphi process to prioritize the panelists’ recommendations into a preliminary checklist.

Checklists can be effective in improving healthcare processes and patient safety [45] by serving at least two purposes: (1) jogging the clinician’s memory and (2) "making the minimum explicit" [46]. But a checklist per se is a weak intervention [47] because it does not address barriers specific to the setting in which it is to be used. A checklist can be effective as part of an intervention that creates social connections among clinicians [26, 47], but not when it functions as the whole intervention. For this reason, our implementation strategy will include not just a checklist, but implementation tools and support.

We convened the advisory panel for a one-day, in-person meeting on November 18, 2014. During the meeting, the authors presented the initial checklist and asked panel members to provide feedback and revisions. We presented a variety of archetypal patient cases (for instance, a patient complaining of pain for the first time versus a patient already taking opioids) and asked panel members to discuss how the checklist might need to be adapted to fit different circumstances. The advisory panel has addressed questions such as: How does the checklist need to be adapted for local context (which research suggests is vital to successful implementation) [47]? Who needs to be involved in the implementation process (e.g. clinic medical directors, nurses, administrative staff)?

Teaming panelists who wrote the clinical guideline for opioid prescribing [32] with implementation experts and primary care physicians was designed to produce a clear picture of how implementation should happen. The implementation guide consists of a checklist containing the essential elements of the guideline and an implementation approach/timetable designed to guide local customization of the checklist.

The two systems consultants for this pilot project are both physician faculty members in the University of Wisconsin’s Department of Family Medicine and Community Health (DFMCH) with current clinical practices. Both are board-certified in family medicine and addiction medicine. They have clinical experience with opioid therapy management in accordance with opioid prescribing guidelines, and have first-hand experience of integrating elements of the guideline into clinical workflows (such as treatment agreements and random drug testing).

The systems consultants have received training in systems-engineering fundamentals in sessions taught by past leaders of the NIATx initiative (on June 30 and November 6, 2015). These training sessions have included topics such as fundamentals of quality improvement, what makes a good consultant, understanding the walk-through exercise, creating and facilitating change teams, using tools such as flowcharting and the nominal group technique, and using Plan-Do-Study-Act cycles.

Specific Aim 2 focuses on field-testing the SCS implementation strategy. The systems consultants’ approach to interacting with clinics is modelled after the coaching protocol used in NIATx 200, which included in-person site visits and regular follow-up through phone and email communication. Participating clinics will be paired with a systems consultant to work through the SCS over a 6-month period. Each clinic will designate one clinician to act as a clinic leader in working with the systems consultant. The clinic leader will host two site visits by the systems consultant and communicate with the consultant monthly during the 6-month follow-up consulting period via phone and email. The clinic leader will work with a clinic’s “change team”—a group of 2 to 6 other staff members involved in opioid prescribing to identify and implement systems changes to improve opioid prescribing processes. The consultant’s initial site visit will last about 2 hours. The consultant will start by presenting the latest research on balancing the benefits and risks of long-term opioid use. The visit will include reviewing information gathered by the change leader before the site visit as a result of conducting a walk-through exercise, in which the change leader follows the clinic process for refilling an opioid prescription for a current patient, step-by-step. The visit will also help the change team determine the best course for implementing aspects of the checklist, depending on the clinic’s own workflow. The systems consultant will help synthesize baseline information from the walk-through and other activities and facilitate a brainstorming session with the change team using nominal group technique [28]. The systems consultant and change team will envision how to implement the checklist, assessing any systemic barriers to implementation. The systems consultant and clinic leader will debrief to develop an implementation plan and schedule a second site visit to assess progress. Throughout the intervention period, the systems consultant will help the team implement ideas for change using Plan-Do-Study-Act change cycles [29] and maintain monthly email and phone contact with the clinic leader and change team to monitor implementation progress and offer advice. The systems consultant will also be available to discuss patient care issues (e.g. difficult cases) during monthly phone conferences, with all identifiable patient data removed beforehand.

### Clinic recruitment

Recruitment will focus on primary care clinics that are part of the DFMCH (n = 20). Clinics offering resident training will be excluded (n = 6); one clinic will be excluded from consideration because one of the systems consultants has an active clinical practice there. We selected the remaining 13 clinics as a recruitment pool to enable systematic monitoring of opioid prescribing rates and other clinical data through the clinical data warehouse housed by the DFMCH. The 13 clinics were first grouped into two categories (community vs. regional) and then ranked by the number of patients with long-term opioid prescriptions (defined as 10+ orders in the previous 12 months). Within these two categories, the clinics were paired by “nearest-neighbour” matching based on number of patients with 10+ opioid prescriptions. One pair of clinics will be recruited from the regional group (2/4 clinics eligible) and three pairs will be recruited from the community group (6/9 clinics eligible). Within each pairing, one clinic will be invited to be the intervention clinic by random selection. If that clinic agrees to participate (via a discussion between the systems consultant and the clinic’s medical director), the second clinic in the pairing will be assigned to the control condition. If the first clinic declines to participate, the second clinic in the pair will be invited to be the intervention clinic. If the second clinic agrees to participate, it will be assigned to be the intervention clinic and the first clinic will be assigned to control. If both clinics decline to participate, an alternate clinic will be selected within each category (there are three alternates among the community clinics and two alternate clinics in the regional clinics) until one of the alternate clinics agrees to participate.

Specific Aim 3 uses mixed methods to understand how the strategy worked, including assessments of feasibility and acceptability (costs, ease of recruitment, fidelity to the protocol, physician acceptance) and preliminary effectiveness (degree of checklist implementation, effect of the strategy on variation in opioid prescribing rates and dosing levels). While multiple prescribers (primary care physicians, nurse practitioners, physician assistants) potentially practice within each clinic, the clinic will serve as the primary unit of analysis. Relevant comparisons for study measures will be made in two ways. First, historical data are available for many study measures, permitting time-series analysis of repeated measures to detect changes in a clinic over time (pre-intervention vs. post-intervention). Second, we can compare intervention and control clinics per the matched-pairs design.

### Measures

This proposal uses the RE-AIM model as an organizing evaluation framework [48] to examine the quality, speed and impact of implementing the SCS in primary care settings. RE-AIM assesses implementation in five dimensions: Reach, Effectiveness, Adoption, Implementation, and Maintenance. This evaluation seeks to meet the standards for ‘fully developed use’ of RE-AIM [49] by employing the RE-AIM checklist available at www.re-aim.org. We have, however, chosen to omit one RE-AIM dimension (maintenance at the setting level) because assessing this dimension would require follow-up 6 months after project funding ends. Specific measures for each RE-AIM dimension are presented in Table 1.

**Table 1:** RE-AIM measures

Domain	Measure
Reach	Number and percentage of patients excluded
Reach	Number and percentage of patients served by eligible clinics
Reach	Characteristics of participating patients versus general patient population
Reach	Structured interview with Family Medicine director to qualitatively assess recruitment process
Effectiveness	Number and percentage of patients completing urine drug screens
Effectiveness	Overall rate of opioid prescribing by clinic and provider
Effectiveness	Number and percentage of patients screened for mental health/substance use problems
Effectiveness	Overall rate of opioid/benzodiazepine co-prescribing
Effectiveness	Number and percentage of patients signing pain agreements
Effectiveness	Number and percentage of opioid prescriptions above 120 mg daily morphine equivalent
Effectiveness	Number and percentage of providers who drop out of study at 3 months
Effectiveness	Structured interview with clinic lead to assess satisfaction, effectiveness, and subgroup differences
Adoption (Setting)	Number and percentage of clinics excluded
Adoption (Setting)	Number and percentage of clinics that participate
Adoption (Setting)	Characteristics of participating clinics versus non-participants
Adoption (Staff)	Number and percentage of staff excluded
Adoption (Staff)	Number and percentage of staff who participate
Adoption (Staff)	Characteristics of participating staff versus non-participants
Implementation	Hours of consulting delivered/received per provider
Implementation	Adaptations made to consulting protocol during intervention period
Implementation	Cost of consulting intervention
Implementation	Structured interview with clinic lead to assess consistency of consulting intervention
Maintenance (Indiv.)	Number and percentage of patients completing urine drug screens (6-month follow-up)
Maintenance (Indiv.)	Overall rate of opioid prescribing by clinic and provider (6-month follow-up)
Maintenance (Indiv.)	Number and percentage of patients screened for mental health/substance use problems (6-month follow-up)
Maintenance (Indiv.)	Overall rate of opioid/benzodiazepine co-prescribing (6-month follow-up)
Maintenance (Indiv.)	Number and percentage of patients signing pain agreements (6-month follow-up)
Maintenance (Indiv.)	Number and percentage of opioid prescriptions above 120 mg daily morphine equivalent (6-month follow-up)
Maintenance (Indiv.)	Number and percentage of providers who drop out of study (6-month follow-up)
Maintenance (Indiv.)	Focus group with clinicians who made substantial changes

Source*:* Re-aim.org; Measuring the Use of the RE-AIM Model Dimension Items Checklist.

### Quantitative data collection and analysis

We will access many of our RE-AIM measures by tapping into clinics’ electronic health records via a data warehouse maintained in the DFMCH (e.g. reach data such as number and percentage of patients excluded, effectiveness and maintenance data such as overall rate of opioid prescribing by provider, etc.). The representativeness of the sample (patients, clinics, and staff) will be accessed through administrative databases maintained by the DFMCH. The quantitative analysis will focus primarily on patient-level opioid prescription rates measured in MEDD. Changes in outcomes will be assessed through repeated monthly observations. Data on prescribing will be augmented by other process and outcome measures shown in Table 1.

### Statistical model

To isolate and measure the intervention effect on each measure of interest, we will fit a mixed-effects model to the data. The model will contain a fixed effect for a shared common linear trend; we will test the sensitivity of the results to other non-linear trends. Fixed effects will be included for the impact of the intervention on the measure of interest. Since the intervention activities will be skewed toward the beginning of the intervention period, we will model an increasing cumulative effect that allows the rate of increase to change during the period. That is, we will use a piecewise linear function of the intervention duration with ‘knots’ at the start and midway through the 12-month period. At the end of the intervention period, we will allow for a second linear progression to capture any continuing effect or any regression back to pre-intervention response levels. Other fixed effects will be included for observed characteristics of providers that may have a significant impact on the response variable (e.g. patient/physician ratio). Random effects will be included to allow for correlation among repeated observations within the same clinic, provider, or patient. Auto-correlated model error terms will be included to allow for additional correlation among observations from the same patient in adjacent months. Appropriate transformations of the response variable (e.g. logarithms, square roots, etc.) will be considered to avoid negative fitted values and to better match the frequency of outlying values.

### Cost analysis

Methods and instruments used for cost data collection in the NIATx 200 study [1] will be adapted to assess the costs of administering the SCS. Systems consultants will keep detailed logs of contacts with clinics (based on an online tracking system developed for NIATx 200) to assess staff participation and fidelity to the protocol. We will estimate the cost of the intervention by assessing time spent by systems consultants and clinicians during the implementation phase and multiplying these estimates by appropriate wage rates based on averages available through the Wisconsin Department of Workforce Development. We will add any relevant non-personnel costs, such as travel to site visits, the cost of teleconferencing services, etc.

### Qualitative data collection and analysis

We will follow NIH guidelines for mixed-methods inquiry [50] to complement quantitative assessments of the feasibility, acceptability, quality, and preliminary effectiveness of the SCS. During the intervention, we will use a variety of qualitative methods, including document review, activity logs, debriefing conversations with the system consultants, and observations of interactions between the systems consultants and the clinic change teams. These methods are designed to help us identify the incentives, scheduling, delivery methods, and other processes and structures that will make the SCS useful for participating clinicians and manageable for the systems consultants. Post-intervention focus groups will be used to compare the experience of clinics that changed substantially versus those that did not. We will explore questions such as (1) What kinds of process changes were associated with improvement? (2) What factors helped providers and clinics make changes? (3) What were the barriers to improvement, and how were they addressed? (4) When the intervention did not work well, what was different?

We will apply a combination of directed and traditional content analysis [51] to answer the questions posed above. Our analysis will identify themes in the qualitative data and organize them into a conceptual model that incorporates a priori elements of self-determination theory (e.g. relatedness, competence, autonomy) and diffusion of innovations theory (e.g. homophily), while simultaneously seeking new insights inductively.

Our second objective in this mixed methods component of our research is to assess fidelity to the intervention, defined as: (1) amount of the intervention received (i.e. ‘dose’), (2) adherence to the protocol, and (3) quality of intervention delivery [52]. Assessing the dose of intervention received will rely on quantitative data (the number of intervention hours delivered to clinic staff) obtained through logs kept by the systems consultants. For adherence, we will review the planned protocol with clinicians and document adaptations made to it at each site. To assess quality, we will examine both quantitative data on clinic-wide practices and focus group data on changes in clinicians’ prescribing attitudes and actions.

### Milestones and products of pilot test

The primary goal of this project is to obtain pilot data for a large-scale clinical trial of different approaches to EBP adoption in healthcare. The research will be critical in developing (1) specifics of the guideline to be implemented, and (2) supports provided via the SCS implementation strategy and the systems consultants who deliver it. The protocol will be determined to be feasible if we enroll four intervention clinics, deliver the intended intervention in all four clinics, follow the clinics for a 6-month period, and obtain quantitative and qualitative data from participating clinicians. Acceptability will be gauged by assessing fidelity to the protocol and analysing qualitative data collected by the research team. We will continue to track effectiveness measures through the end of the 3-year grant period using de-identified electronic health records (e.g. opioid prescribing rate by clinician, number of treatment agreements signed) to assess the preliminary effectiveness of the SCS on clinical practice. With eight clinics reporting data (four intervention and four control clinics), we will be able to obtain baseline estimates of means and standard deviations for RE-AIM outcomes and point estimates of treatment effect sizes, thereby equipping us to conduct an accurate power calculation for the follow-up randomized trial.

### Potential problems and alternative strategies

We are confident we can recruit four intervention clinics from our recruitment pool based on successful prior collaboration between the University of Wisconsin Departments of Industrial & Systems Engineering and DFMCH. If we are unable to recruit four intervention clinics from the 13 family medicine clinics we are targeting, we will extend our recruitment efforts to the six DFMCH community clinics that train residents (and exclude from analysis clinicians who are residents).

Prescribing opioids for chronic non-cancer pain is complex and controversial. The potential exists for physicians to be reluctant to participate in the study because they fear having their opioid prescribing practices scrutinized. We will employ several strategies to address this: (1) the primary unit of analysis will be the clinic; (2) individual prescribers will not be identified in study databases, and every precaution will be taken to maintain confidentiality; (3) the systems consultant will emphasize that aspects of pain management lack an adequate evidence base, and that the purpose of the study (and the larger trial) will be to provide the evidence needed to improve opioid prescribing practices; and (4) the presence of national experts involved in the research may motivate physicians and clinics to participate.

### Limitations

As pilot implementation research, we are focused on assessing the feasibility, acceptability, and preliminary effectiveness of the implementation strategy and not long-term patient-level outcomes. Secular trends in opioid prescribing could make it difficult to gauge the effectiveness of the SCS (i.e. as public awareness of the opioid crisis increases, rates of prescribing may fall). We will partially control for this limitation by comparing trends in intervention versus control clinics.

### Future directions

As outlined earlier, we intend to conduct a large-scale RCT to test the costs and effects of different approaches to EBP adoption in healthcare, including audit/feedback, academic detailing, and the SCS. We expect that some of the physicians who participate in this pilot research might themselves become systems consultants; indeed, 9 of the 17 coaches in the NIATx 200 study were former change leaders in addiction treatment clinics who went on to coach their peers.

### Current status

When this manuscript was submitted, the research team was transitioning from Study Aim 1 (guideline translation and consultant training) to Study Aim 2 (field testing the implementation strategy). Results will be published as they become available.

### Ethics approval

The study protocol has been designated minimal risk and approved by the University of Wisconsin’s Health Sciences Institutional Review Board (2015-0280-CP003).

## Discussion

Given that this is pilot research and that implementation is always dynamic, elements of the research design reported in this paper will undoubtedly change after it is published. One illustration of this dynamic pertains to the naming of the implementation strategy. In the grant proposal, we named the strategy “NIATx-VOP” (i.e. Network for the Improvement of Addiction Treatment – Variation in Opioid Prescribing). However, initial discussions with community-based primary care physicians highlighted that the name ‘NIATx’ has been primarily identified with quality improvement in specialty addiction treatment and holds little currency in primary care. Further, primary care physicians told us that “doctors don’t like to be coached.” In response, we have tentatively changed the name of the implementation strategy from ‘NIATx-VOP’ to ‘systems consulting strategy’ and the word ‘coach’ to ‘systems consultant’. As pointed out by Proctor et al. [53], naming implementation strategies is an important aspect of specifying them; thus, it may be that the name assigned to the strategy will change again based on feedback gathered during our fieldwork.

## Conclusion

Developing an effective strategy for implementing evidence-based practices for opioid prescribing in primary care is an urgent matter, given that primary care is the origin of most opioid prescriptions, the rate at which patients taking opioids for chronic pain become addicted, and the increasing number of lives compromised or ended by opioid abuse and addiction. The novel implementation strategy proposed here (guideline translation and peer consulting using tools of systems engineering) could readily be generalized to other EBPs, and holds the potential to exert a powerful and sustained impact not only on opioid prescribing but perhaps on the implementation of EBPs throughout healthcare. Such a strategy could potentially be used for other evidence-based practices, shortening the famously long time it takes for such practices to be implemented in healthcare.
